# Supplementary figures and images for: Specific and comprehensive genetic targeting reveals brain-wide distribution and synaptic input patterns of GABAergic axo-axonic interneurons
Source: bioRxiv. 2024 Mar 25:2023.11.07.566059. Originally published 2023 Nov 7. Preprint. [Version 2] doi: 10.1101/2023.11.07.566059 (PMC10659298; doi:10.1101/2023.11.07.566059)

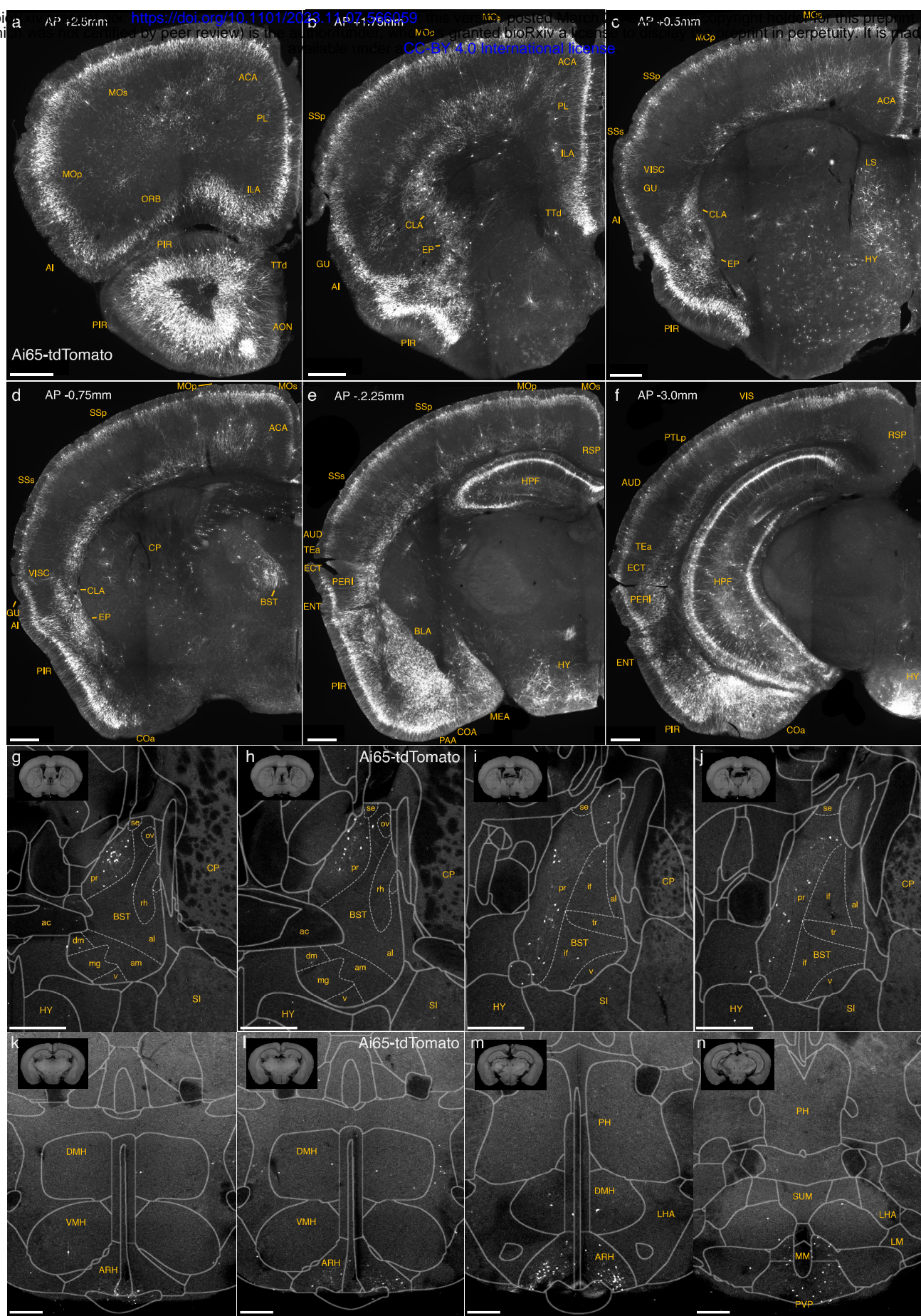

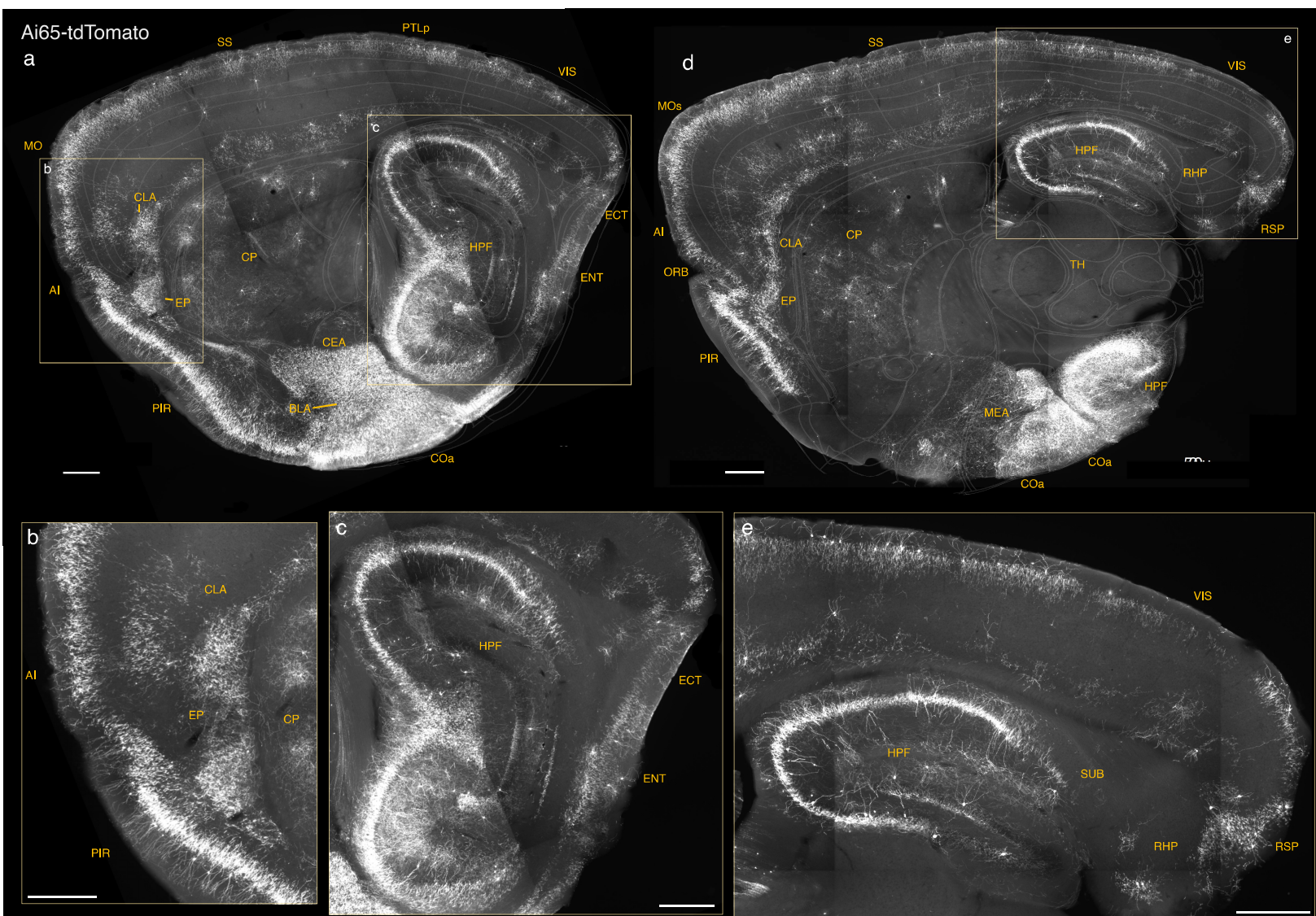

Fig 1 Suppl 2

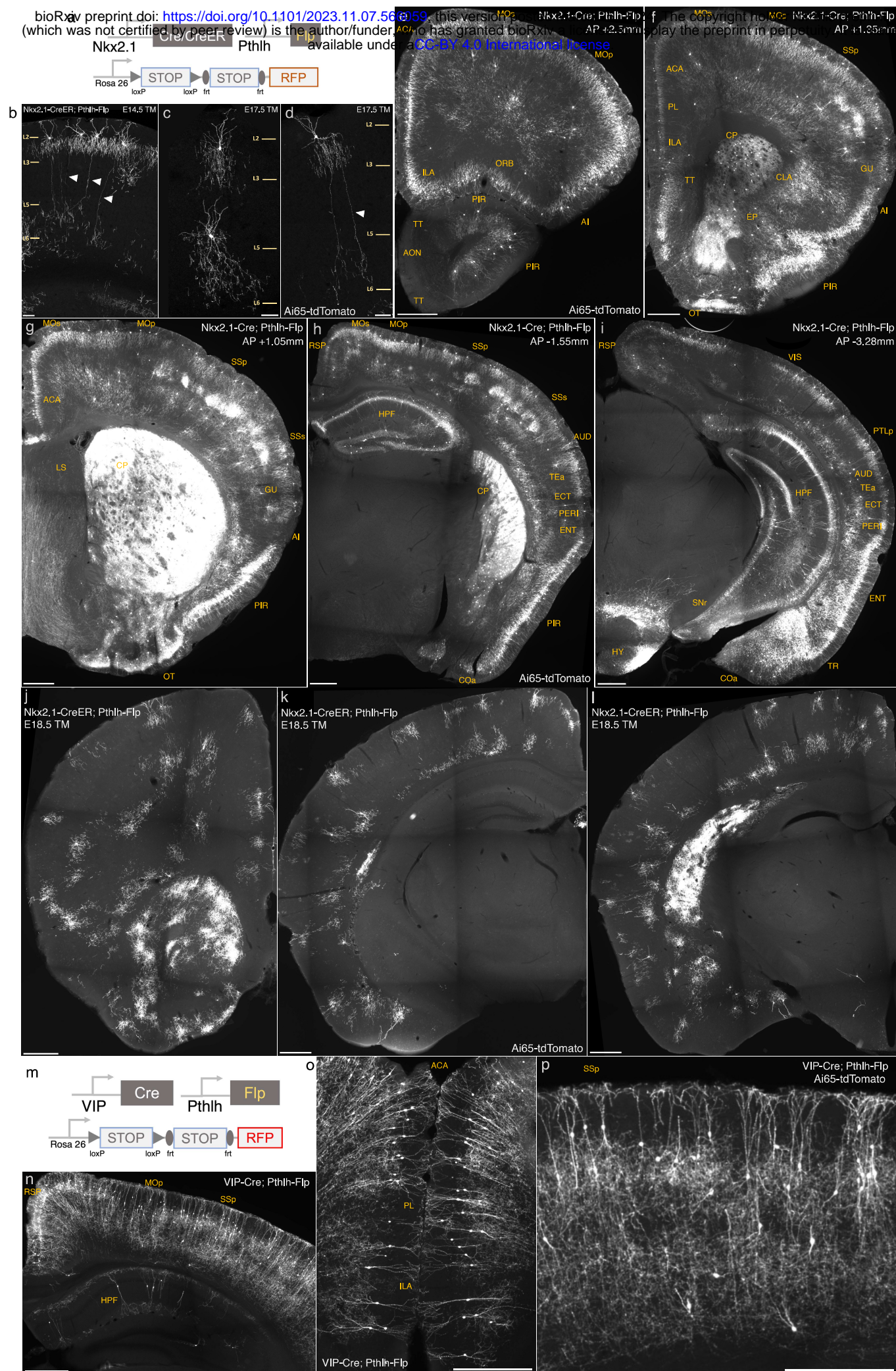

Fig 1 Suppl 3

**Fig 2**  
**Suppl**  
**Fig 1**

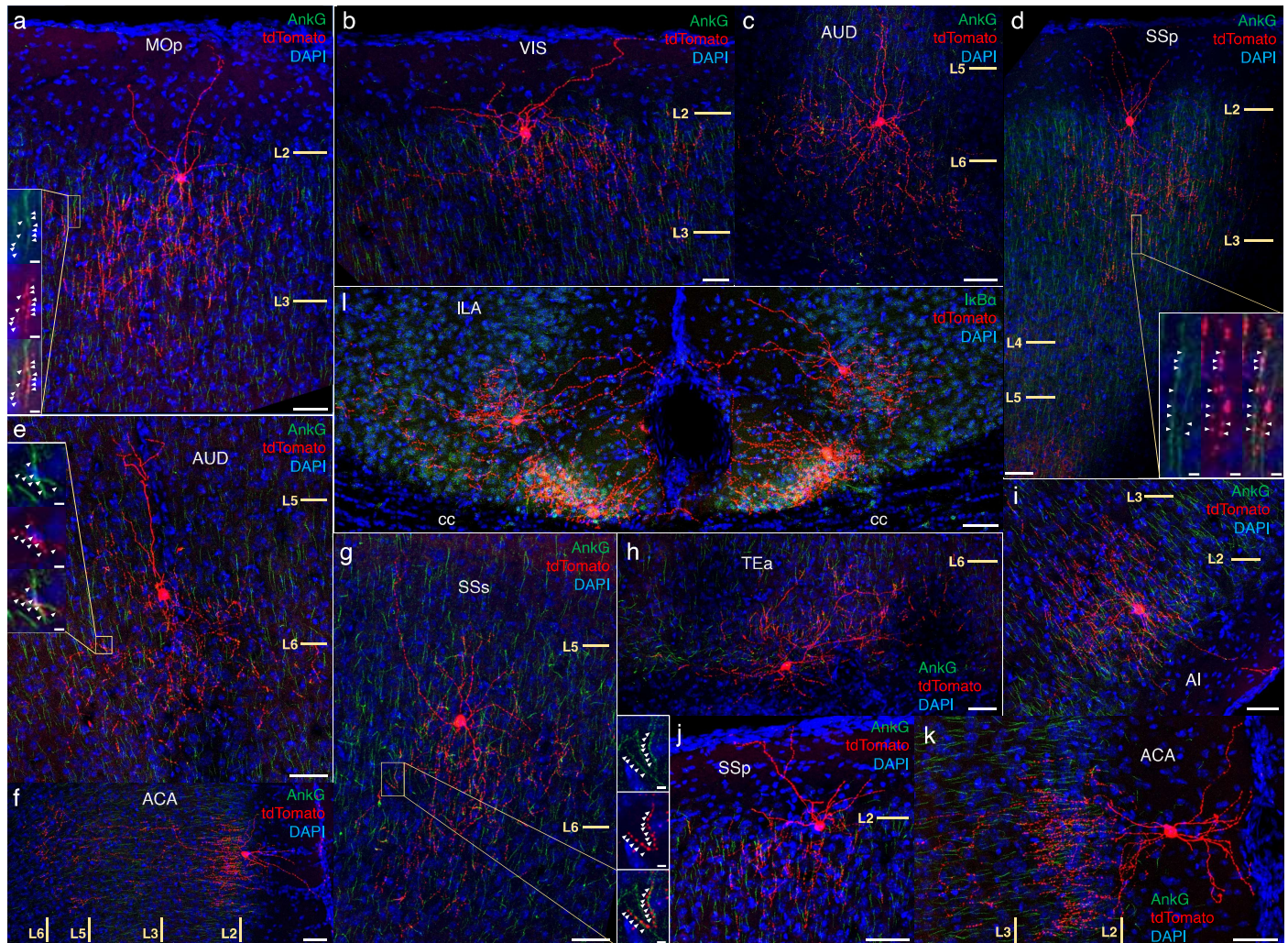

Fig 7 Suppl 1

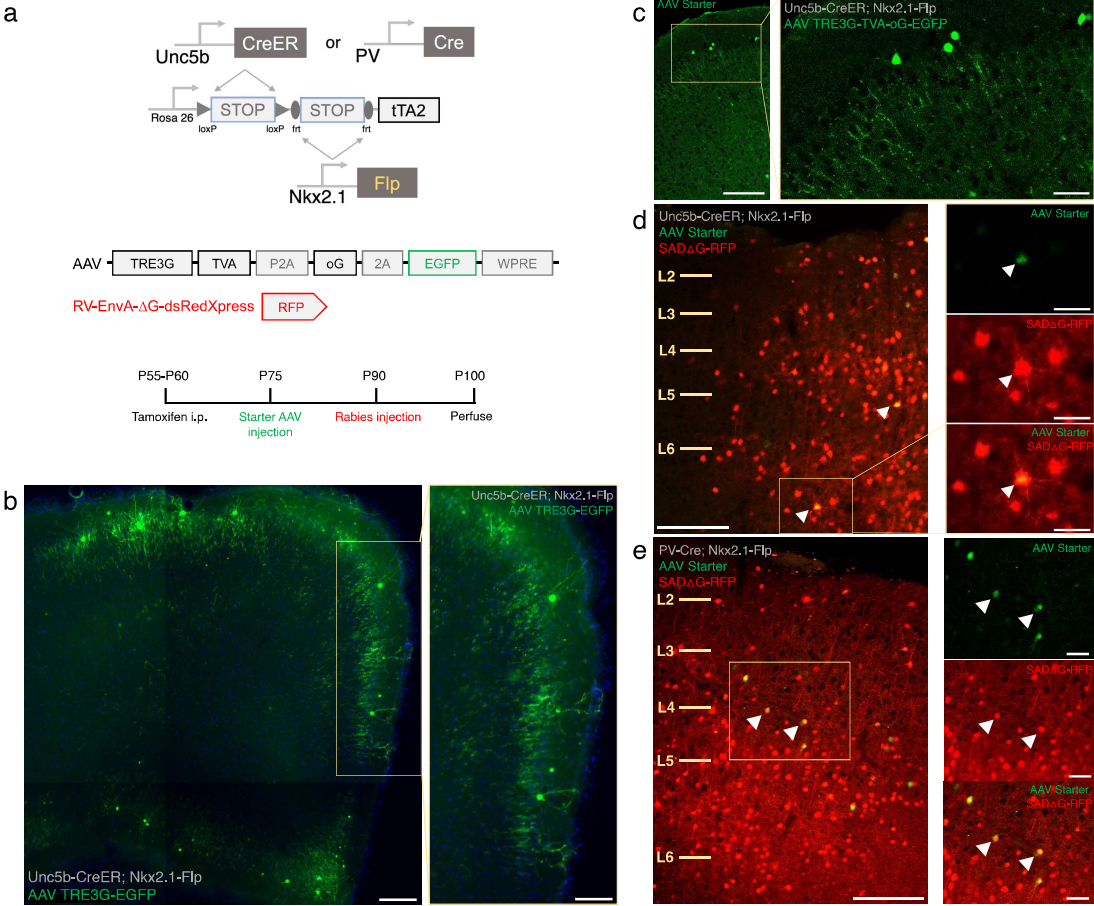

Supplement: Supplement 2 — Figure 1 Supplement Figure 1. Dense labelling of AACs across brain regions in a Unc5b-CreER;Nkx2.1-Flp;Ai65 mouse. (a-f) Additional representative coronal sections showing dense Ai65 expression patterns at specified anterior-posterior coordinates (from Bregma). Note labeling in cerebral cortex (a-f); hippocampus (e-f); olfactory centers, including piriform, anterior olfactory nucleus, and tenia tecta dorsal (PIR, AON, TTd) (a-b); agranular insula, claustrum, and endopiriform nucleus (AI, CLA, EP) (b-c); amygdaloid complex and extended amygdala (COA, BLA, MeA) (d-f); Bed nuclei of the stria terminalis (BST) (d); and hypothalamus (HYP) (e-f). (Scale bars, 500μm.) (g-j) Ai65-labelled pAACs in the bed nuclei of the stria terminalis (BST) are largely confined to the principal nucleus (BSTpr), with pAACs clustering more densely in anterior BSTpr (g-h) compared to more posterior segments (i-j). (k-n) Ai65-labelled pAACs in the hypothalamus are densest in the ventral-medially area of the arcuate nucleus (ARH), with highest density in caudal portions (k-m) and more posteriorly in the paraventricular hypothalamic nuclei (PVP) (n). pAACs are also present sparsely in the dorsal-medial nucleus of the hypothalamus (DMH) (k-m). Figure 1 Supplement Figure 2. Additional sagittal sections of Unc5b-CreER; Nkx2.1-Flp; Ai65 mice highlighting dense pAACs in medial, lateral, and ventral pallium-derived structures. (a-c) A lateral sagittal section showing the lPAL-derived anterior insular (AI), claustrum (CLA), endopiriform (EP) (magnified in b) and mPAL-derived hippocampal formation (magnified in c). Note the single-banded orientation of hippocampal (c) and insular (b) AACs that is absent in more inferior lPAL structures (b). (d-e), A more medial sagittal section showing dorsal and ventral hippocampus and retrohippocampal region (RHP) (d), retrohippocampus, and visual and retrosplenial cortex (e). Compare the dual-banded AAC distribution in upper and deeper cortical layers to the single-b [file NIHPP2023.11.07.566059v2-supplement-2.pdf]
