# Supplementary material for: Specific and comprehensive genetic targeting reveals brain-wide distribution and synaptic input patterns of GABAergic axo-axonic interneurons
Source: bioRxiv. 2023 Nov 7:2023.11.07.566059. Preprint. [Version 1] doi: 10.1101/2023.11.07.566059 (PMC10659298; doi:10.1101/2023.11.07.566059)

bioRxiv preprint doi: https://doi.org/10.1101/2023.11.07.566059; this version posted November 8, 2023. The copyright holder for this preprint (which was not certified by peer review) is the author/funder, who has granted bioRxiv a license to display the preprint in perpetuity. It is made available under aCC-BY 4.0 International license.

Figure 1 Supplement Figure 1

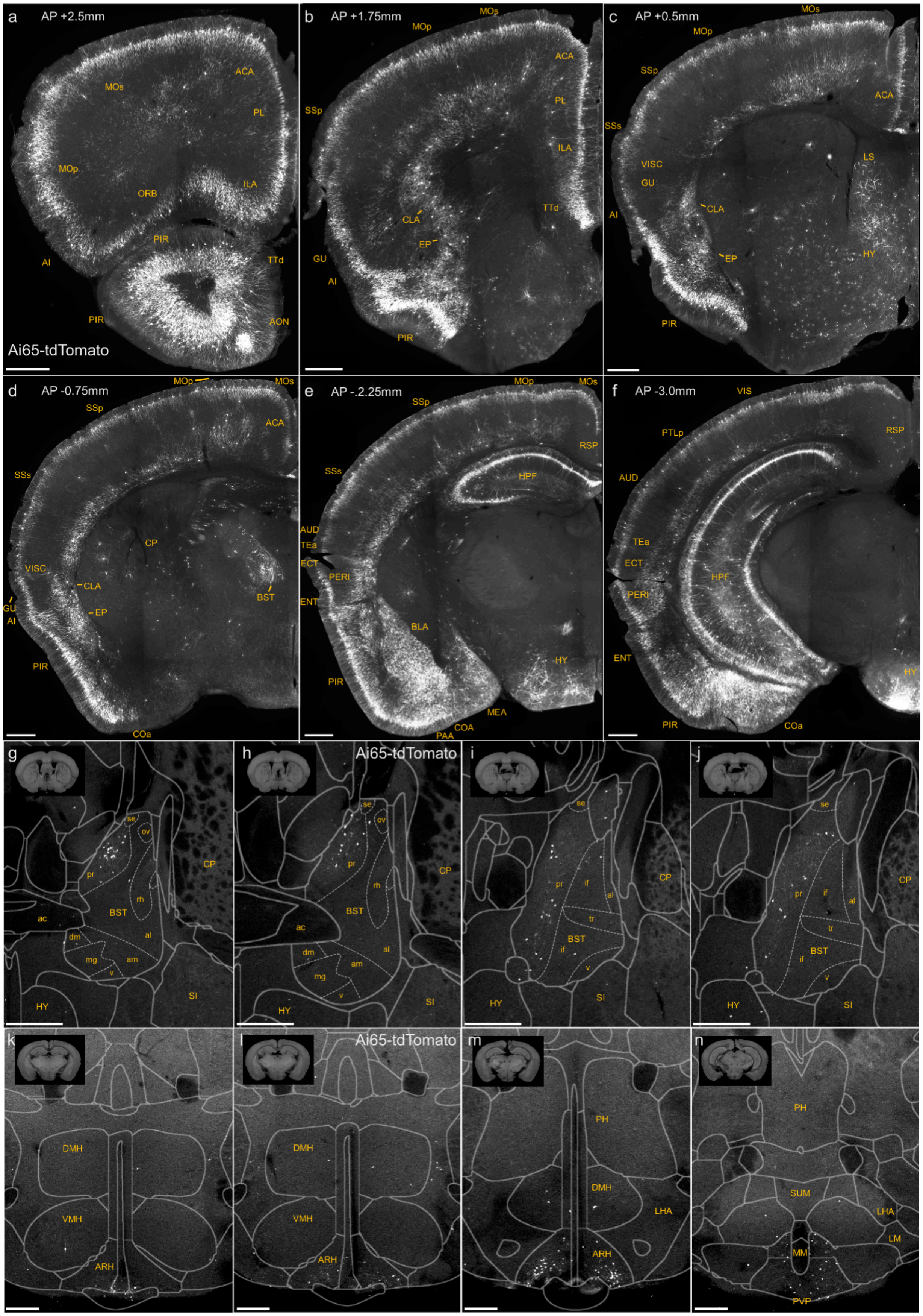

bioRxiv preprint doi: https://doi.org/10.1101/2023.11.07.566059; this version posted November 8, 2023. The copyright holder for this preprint (which was not certified by peer review) is the author/funder, who has granted bioRxiv a license to display the preprint in perpetuity. It is made available under aCC-BY 4.0 International license.

Figure 1 Supplement Figure 2

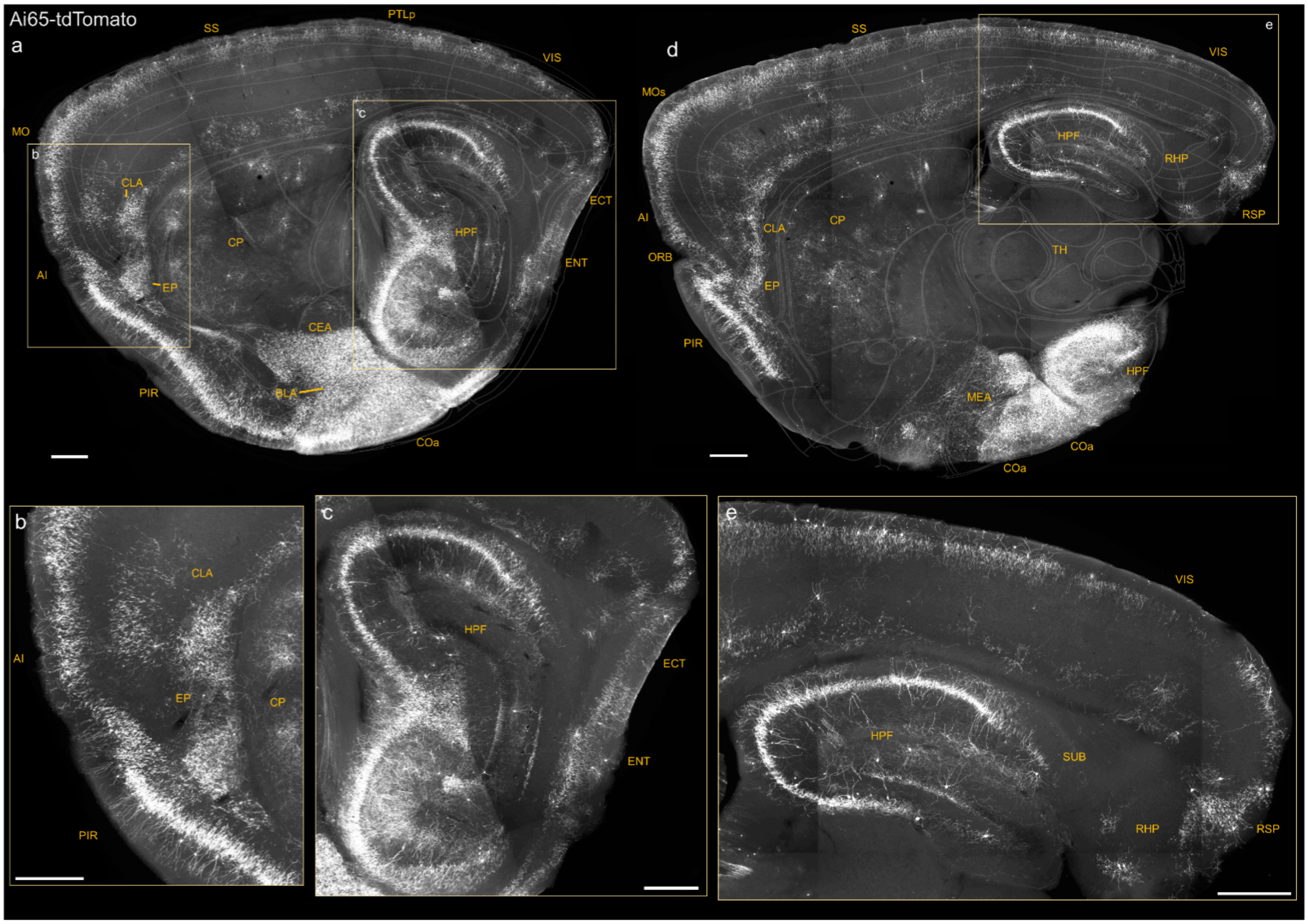

bioRxiv preprint doi: https://doi.org/10.1101/2023.11.07.566059; this version posted November 8, 2023. The copyright holder for this preprint (which was not certified by peer review) is the author/funder, who has granted bioRxiv a license to display the preprint in perpetuity. It is made available under aCC-BY 4.0 International license.

Figure 1 Supplement Figure 3

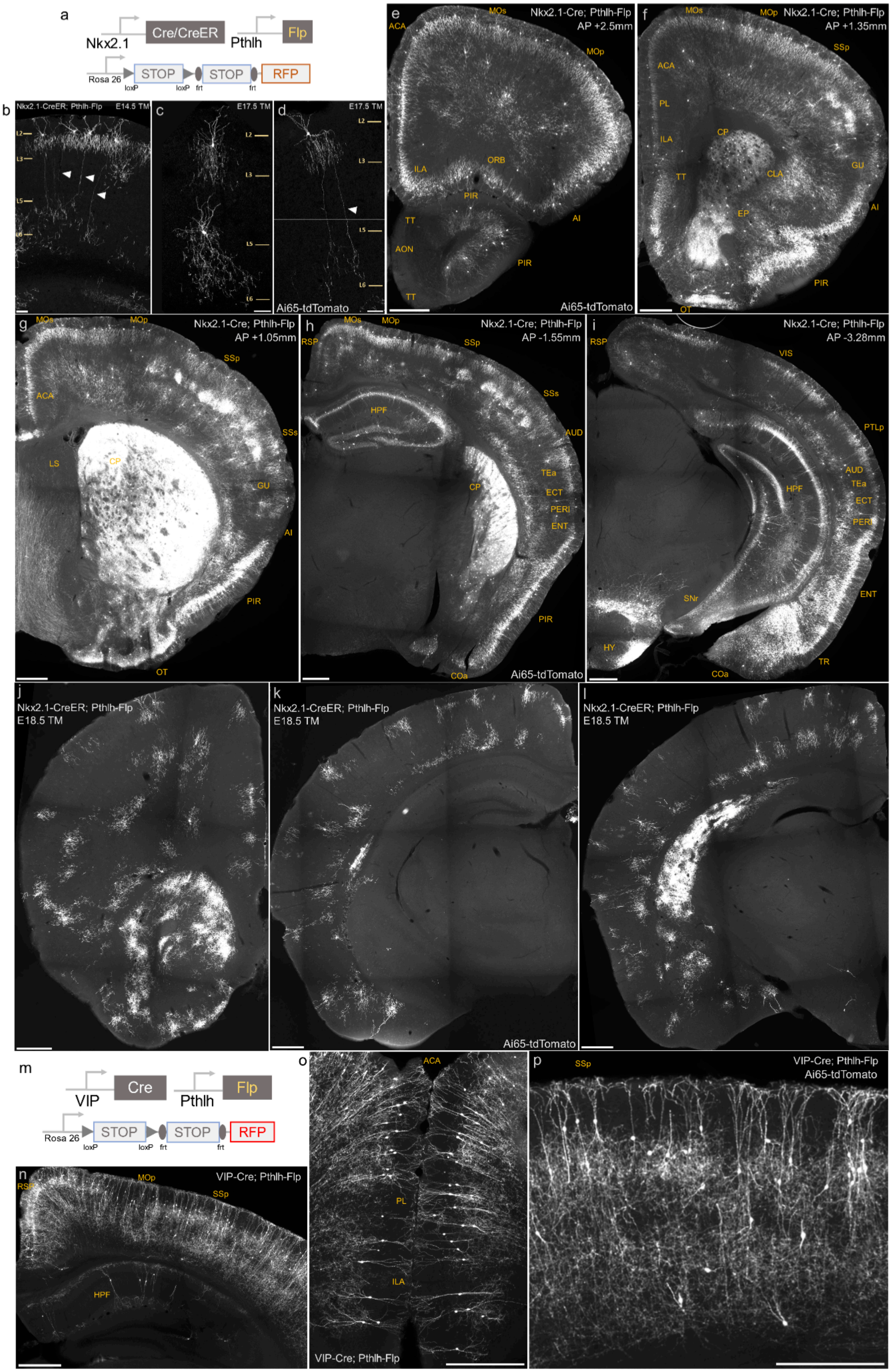

bioRxiv preprint doi: https://doi.org/10.1101/2023.11.07.566059; this version posted November 8, 2023. The copyright holder for this preprint (which was not certified by peer review) is the author/funder, who has granted bioRxiv a license to display the preprint in perpetuity. It is made available under aCC-BY 4.0 International license.

Figure 2 Supplement Figure 1

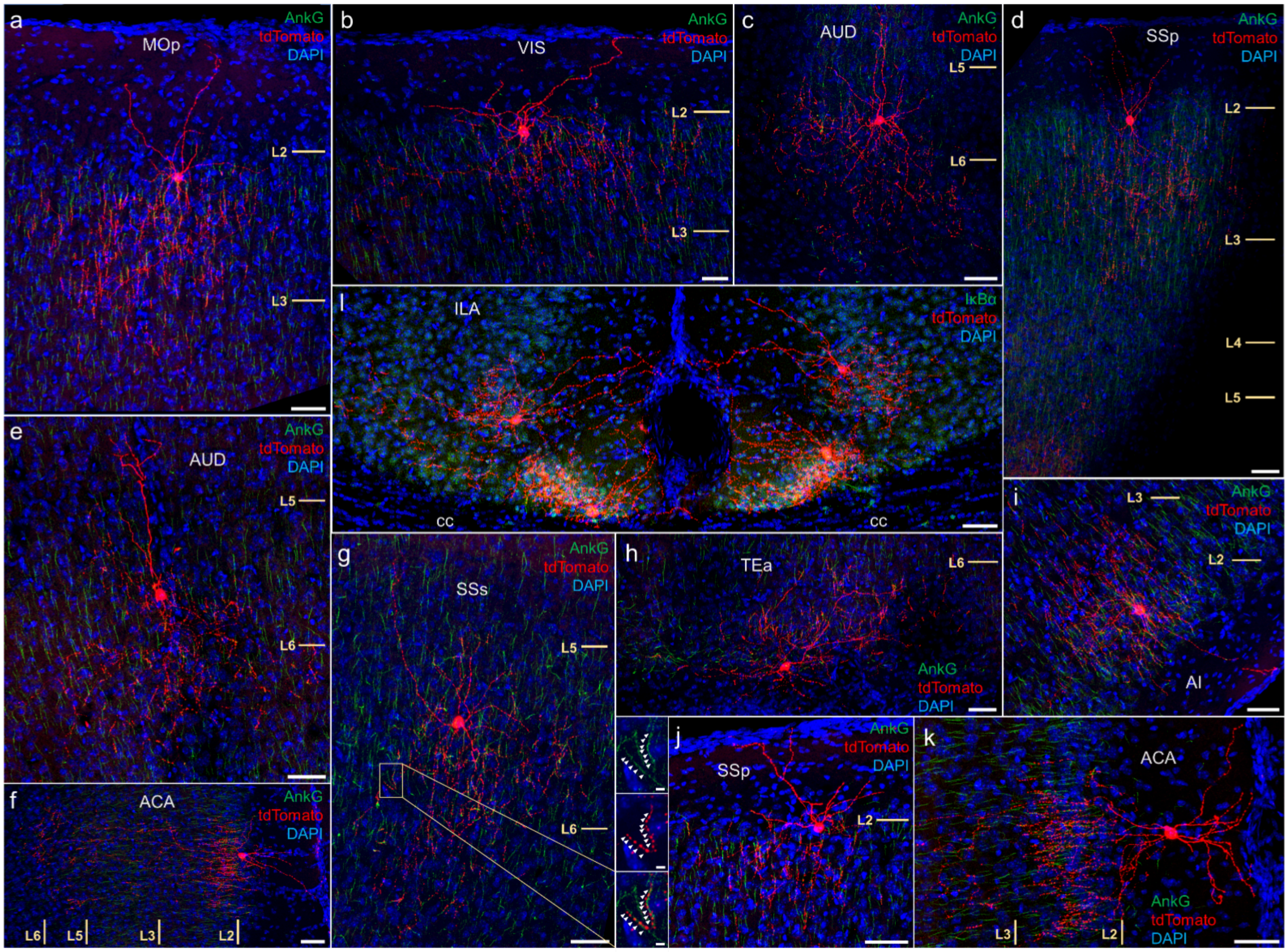

bioRxiv preprint doi: https://doi.org/10.1101/2023.11.07.566059; this version posted November 8, 2023. The copyright holder for this preprint (which was not certified by peer review) is the author/funder, who has granted bioRxiv a license to display the preprint in perpetuity. It is made available under aCC-BY 4.0 International license.

Figure 7 Supplement Figure 1

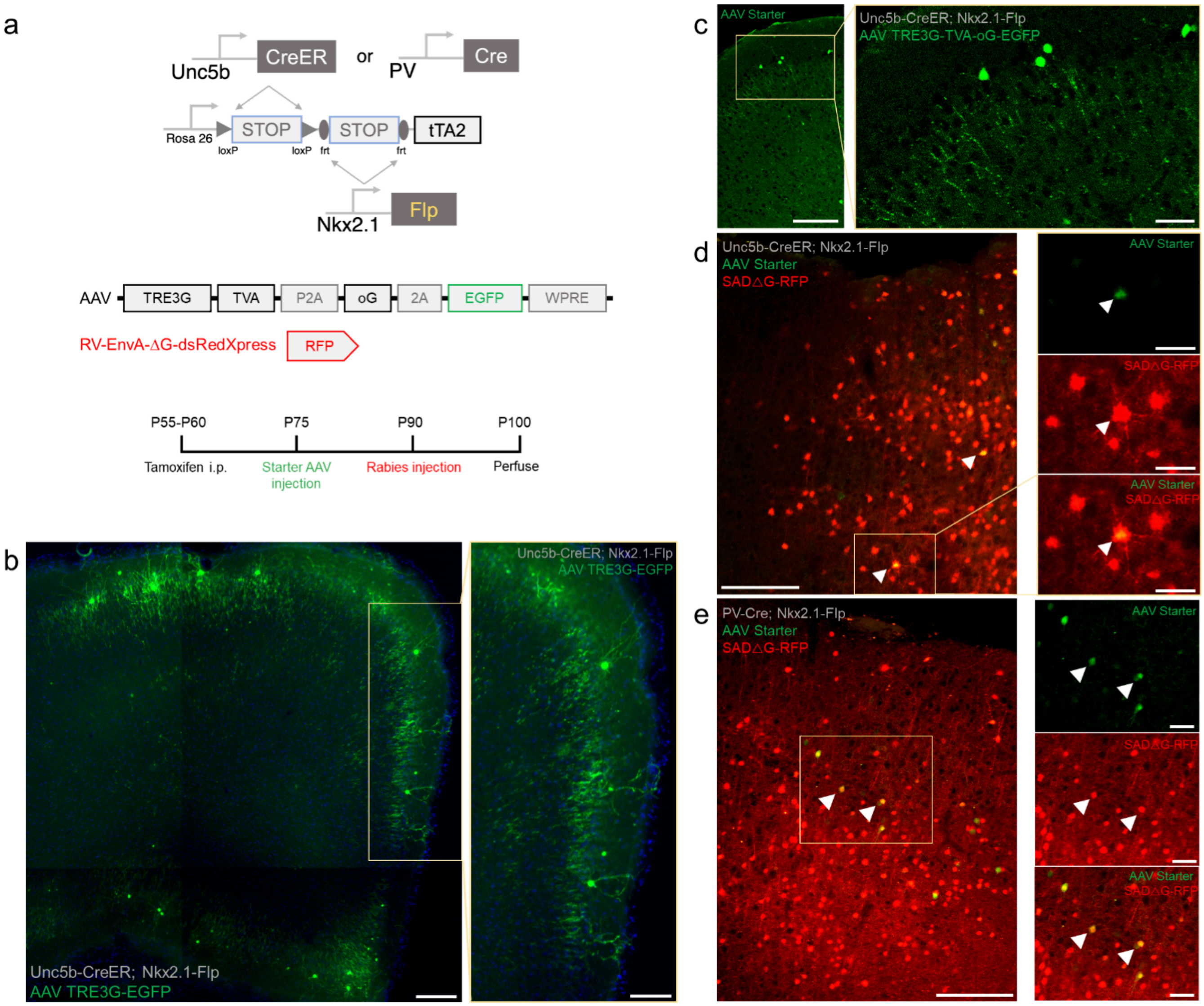

Supplement: Supplementary file 1 [file NIHPP2023.11.07.566059v1-supplement-1.pdf]
